# A novel genetic variant in *DNAI2* detected by custom gene panel in a newborn with Primary Ciliary Dyskinesia: case report

**DOI:** 10.1186/s12881-020-01160-5

**Published:** 2020-11-10

**Authors:** Maria Santa Rocca, Gioia Piatti, Angela Michelucci, Raffaella Guazzo, Veronica Bertini, Cinzia Vinanzi, Maria Adelaide Caligo, Angelo Valetto, Carlo Foresta

**Affiliations:** 1 Department of Medicine, University of Padova, Via Giustiniani, 2, 35128 Padova, Italy; 2 Unit of Bronchopneumology, Department of Pathophysiology and Transplantation, Fondazione IRCCS Ca’ Granda, Ospedale Maggiore Policlinico, University of Milan, Milan, Italy; 3 Laboratory of Molecular Genetics, University Hospital of Pisa, Pisa, Italy; 4 Unit of Pathological Anatomy, Department of Medical Biotechnology, University of Siena, Siena, Italy; 5 Laboratory of Cytogenetics, University Hospital of Pisa, Pisa, Italy

**Keywords:** Primary ciliary dyskinesia, *DNAI2*, Outer dynein arm, PCD genetic panel, Normal pressure hydrocephalus

## Abstract

**Background:**

Primary ciliary dyskinesia (PCD) is a highly heterogeneous genetic disorder caused by defects in motile cilia. The hallmark features of PCD are the chronic infections of the respiratory tract, moreover, clinical manifestations include also laterality defects and risk of male infertility.

Clinical phenotypes of PCD are the result of mutations in genes encoding components of axonema or factors involved in axonemal assembly. Recent studies have identified over 45 PCD-associated genes, therefore, molecular analysis represents a powerful diagnostic tool to confirm and uncover new genetic causes of this rare disease.

**Case presentation:**

Here, we describe a female infant of Moroccan origin with normal pressure hydrocephalus (NPH) in addition to most common PCD symptoms. Transmission Electron Microscopy (TEM) and molecular tests, such as a Next generation Sequencing panel and a custom array CGH, were performed for diagnosis of PCD. TEM revealed outer dynein arm (ODA) defects, whilst molecular analyses detected a novel 6,9 kb microdeletion in *DNAI2* gene.

**Conclusions:**

Since *DNAI2* mutations are very rare, this case report contributes to better delineate the important role of DNAI2 as causative of PCD phenotype, suggesting, furthermore, that the variations in *DNAI2* may be as a new genetic risk factor for NPH. Indeed, although the association of hydrocephalus with PCD has been well documented, however, only a small number of human patients show this defect.

Furthermore, this study highlights the importance of high-throughput technologies in advancing our understanding of heterogeneous genetic disorders.

**Supplementary Information:**

The online version contains supplementary material available at 10.1186/s12881-020-01160-5.

## Background

Primary ciliary dyskinesia (PCD) (MIM: 244400) is a rare heterogeneous disorder caused by dysfunction of motile cilia, resulting in recurrent respiratory infections due to impaired mucociliary clearance. The disease is predominantly caused by mutations in genes encoding for the ciliary axonemal motor proteins that regulate ciliary beat.

PCD is mainly transmitted in an autosomal recessive pattern and has a prevalence of 1 in 10,000–20,000 individuals [24]. Typical clinical manifestations of this disorder include neonatal respiratory distress, chronic rhinosinusitis, hearing impairment, development of bronchiectasis, male infertility and situs abnormalities (predominantly situs inversus, rarely situs ambiguous) occurring in about 50% of cases. However, less common clinical manifestations have been also described in PCD patients, such as oesophageal disease, biliary atresia, complex congenital heart disease and hydrocephalus [2, 6, 19].

Since many symptoms of PCD can overlap with other most common respiratory diseases, this leads to an underestimation of the real prevalence of this disorder [3, 31].

Furthermore, since PCD lacks of a “gold standard” test the diagnosis is usually based on a combination of tests: nasal nitric oxide (nNO) measurement, high-speed video microscopy analysis (HSVMA), TEM and genetic tests [30].

The heterogeneity of the clinical presentations in PCD patients reflects the genetic heterogeneity of this disorder; moreover, heterogeneity may also exist even between patients with the same genetic defect [34]. To date, indeed, mutations in approximately 45 genes have been linked to PCD [9, 35] and genetic test is currently able to identify 70–80% of PCD cases [7].

To establish the genetic diagnosis, non-ambiguous bi-allelic mutations in autosomal recessive PCD should be identified [22]. Among all genetic mutations, nearly 15–20% of all PCD patients results from mutations within Dynein Axonemal Heavy Chain 5 (*DNAH5*) gene (MIM: 603335) [10, 27] causing outer dynein arm (ODA) defects [14]; *DNAH5* and Dynein Axonemal Intermediate Chain 1 *(DNAI1)* mutations are the most frequent mutations encountered in correlation with ODA defects, ~ 50% and ~ 10% of cases, respectively [23]; mutations in other genes are much less frequent.

Dynein Axonemal Intermediate Chain 2 (*DNAI2*) is a component of ODA complex and is essential for the assembly of this multimeric complex. The protein is encoded by *DNAI2* gene (MIM: 605483) consisting of 14 exons and mapping to 17q25; mutations in *DNAI2* gene are a rare cause of PCD with associated ODA defects at ciliary ultrastructure analysis (2–4%) [21].

Here, we describe the case of a four-month-old female infant carrying a novel homozygous deletion in *DNAI2*, and hypothesize that the typical signs of PCD of this patient, in addition to a rare clinical manifestation associated to PCD such as hydrocephalus, are the result of the identified genetic variation. Furthermore, we support the application of high-throughput molecular techniques for the analysis of patients affected by this rare disorder.

## Case presentation

### Clinical characteristics

The patient was a female second-born child of healthy non-consanguineous Moroccan parents with an unremarkable family history.

Foetal ultrasonography (US) performed at the second trimester of gestation showed brain ventriculomegaly and *situs inversus*. The proband was born by spontaneous delivery at 41 week of gestation. The day after the birth, she showed a mild polypnoea requiring oxygen supplementation because of a low HbO_2_ saturation (< 90%). At third day of age, the newborn showed stable cardiorespiratory parameters and oxygen was discontinued after one week; no ventilatory support was necessary. A lobar collapse was observed on chest X-ray.

Antibiotic therapy was requested to reduce the respiratory inflammation pointed out from high CRP (C-Reactive Protein) and white blood cell (WBC) count values, but microbiological tests on blood and tracheal aspirate resulted negative. The patient remained hospitalized for two weeks and dismissed from hospital with diagnosis of brain ventriculomegaly, *situs inversus* and patency of foramen ovale.

A brain MRI carried out on the fifth day of life detected an enlargement of III ventricle and lateral ventricles and NPH. Severe enlargement of lateral ventricles was confirmed with echography at the second month of age. The patient was followed-up during the first months of life and only monitored with echography and by neurological visits, including monitoring of head circumference measure. The neurological examination at third month of life found a girl reactive, without asymmetries, with good control of the head and who began to control the trunk. A lumbar puncture was not performed; no surgical treatment for hydrocephalus were carried out.

Observational examination at fourth month of age did not reveal neurological anomalies.

A karyotype analysis was performed revealing a normal female karyotype (46,XX).

### Diagnostic tests

The study of ciliary motility performed at third month of age did not identify ciliated cells suitable for analysis of ciliary beat frequency and pattern, but showed exclusively inflammatory cells and many bacteria.

Ciliary brushing biopsy was taken from the nasal middle turbinate for ciliary ultrastructure analysis.

At least 50 cross section of cilia from different cells were observed for ultrastructural analysis.

### Genetic testing

DNA was isolated from peripheral blood leukocytes using QIAamp DNA Blood Mini Kit (Qiagen Inc., Hilden, Germany) for molecular analysis. We carried out a custom Next Generation Sequencing (NGS) using a panel including the coding exons and flanking regions of 36 genes known to be involved in PCD (Additional file 1) and sequenced on a MiSeq sequencer (Illumina, San Diego, CA, USA). Read alignment to reference genome (hg19), variant calling and annotation were performed with the Agilent SureCall software. The list of putative variants obtained were SNVs and small insertions and deletions with respect to a reference genome. Finally, the sequencing coverage of each exons was analysed in detail using IGV (Integrative Genomic Viewer) visualization tool.

For array Comparative Genomic Hybridization (aCGH) analysis, 200 ng of DNA from the patient (test sample) and a human reference female DNA (cat 5190–3797) of Agilent kit were differentially labelled with Cy5-dCTP or with Cy3-dCTP using random primer labelling according to manufacturer’s protocol (Agilent). The array CGH was performed on a customized 60 K SurePrintG3 Human CGH Microarray (Agilent), enriched in the genes included in NGS panel. The slide were washed and scanned using the Agilent scanner and the identification of individual spots on scanned arrays and quality slide evaluation was performed with the Agilent dedicated software (Feature Extraction, Agilent). For these genes, the overall median probe spacing is about 1.8 Kb. Copy Number Variations were identified with Cytogenomics 3.0.6.6. (Agilent), using the ADM-2 (Aberration Detection Method-2) algorithm. We analysed all the CNVs, independently of their absolute size; they were compared to those reported in the http://dgv.tcag.ca/variation.

**Fig. 1:**
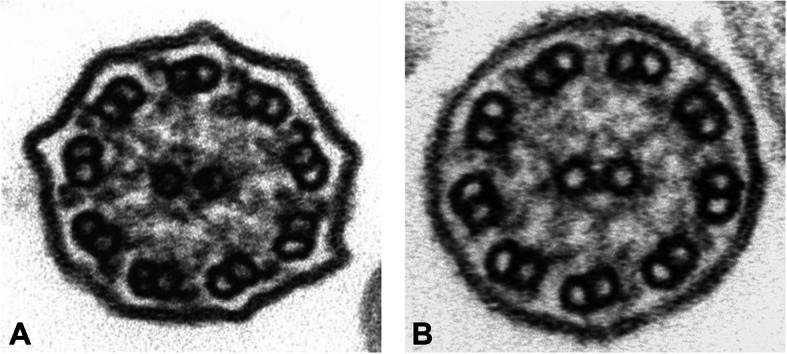
**a** Ultrastructure of a normal cilium from nasal epithelium of a healthy subject showing nine peripheral pairs of microtubules with outer and inner dynein arms clearly visible (100.000 x). **b** Representative transmission electron micrograph of a cross-sectioned cilium from the patient demonstrating the absence of outer dynein arms (100.000x)

TEM highlighted the absence or shortened outer arm dyneins in 100% of analysed cells (Fig. 1).

**Fig. 2:**
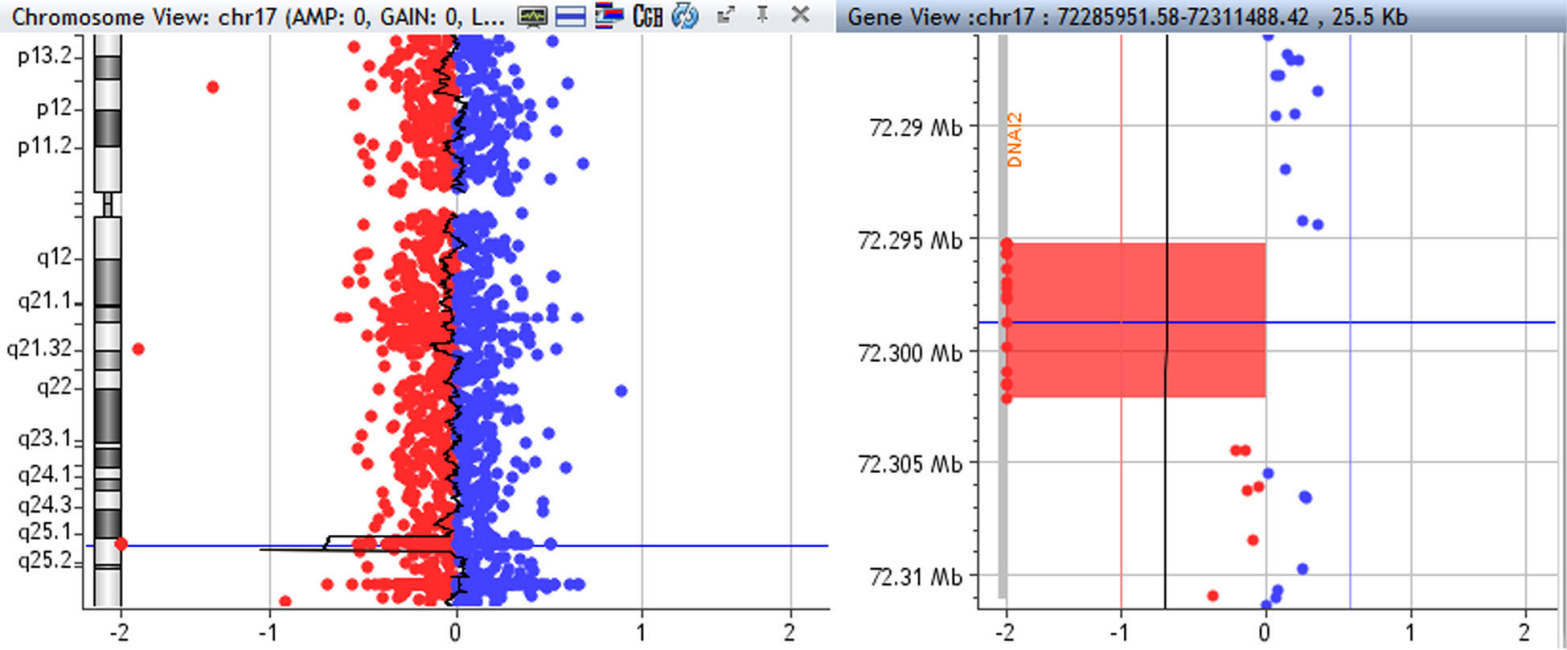
Chromosome 17 array profile (left); highlight of the homozygous deleted region, involving the *DNAI2* gene (right)

Sequencing by NGS panel identified a homozygous deletion of 7–9 exons in *DNAI2* gene (chr17: 72.295.857–72.301.581; hg19). The deletion was confirmed by custom aCGH that detected a 6,9 kb homozygous deletion in *DNAI2* gene, starting from position 72.295.232 to position 72.302.209 (GRCh37/hg19) in 17q25.1: arr 17q25.1(72.295.232–72.302.209) × 1 (Fig. 2).

Unfortunately, the unavailability of both parental DNA did not allow us to determine inheritance pattern of the identified deletion.

## Discussion and conclusions

PCD is a rare genetically disorder characterized by chronic respiratory disease, infertility and situs anomalies occurring in about 50% of cases as result of defective motile cilia. Cilia are specialized organelles that extend from the cellular surface of respiratory epithelium, ependymal cells, gonads and embryonic node [35]. Mutations in any protein involved in cilia assembly, structure or function could cause disease and to date a considerable number of genes have been implicated in this disorder [9].

Diagnosis of PCD is often delayed or missed completely, especially when organs symmetry is correct. An European survey on paediatric cases showed a median age of diagnosis of 5.3 years, lower in children with *situs inversus* (3.5 years vs 5.8 years) [18].

Under-diagnosis or late-diagnosis of PCD likely could contribute to increase the risk of a progressive decline in lung function in these patients [26].

Although the assessment of ciliary ultrastructure by TEM was previously considered the gold standard for PCD diagnosis [17], it is known that up to 30% of PCD cases can have a normal ciliary structure appearance [16], since they have mutations that are not associated with ultrastructural defects [4, 22]. Therefore, over last years, advances in genetic testing and molecular biology, have improved knowledge on this hereditary rare disease. The molecular analysis, in combination with other diagnostic tests, such as ultrastructural microscopy, high-speed video microscopy and nNO determination, represents, hence, an important diagnostic tool to confirm PCD [20].

Furthermore, based on the large number of PCD-causing genes, it has become evident that the application of new high-throughput technologies can simplify the genotype-phenotype correlation and complement ciliary analysis [13]. To date, mutations in over 45 genes have been identified as causative of PCD [9]. Such mutations are in genes encoding axonemal motor proteins, structural and regulatory elements, and cytoplasmic proteins, that are proteins involved in assembly and pre-assembly of ciliary elements [8]. However, the identification of bi-allelic pathogenic variants in known PCD-associated genes should be enough to give a diagnosis of PCD [9].

In the present study, we report the case of a four-month-old female infant carrying a novel homozygous deletion within *DNAI2* gene detected by NGS sequencing and confirmed by custom aCGH.

DNAI2 is a member of the ODA complex and its gene, *DNAI2*, results mutated in approximately 2 to 4% of patients with PCD [13, 14, 21].

To best our acknowledgement, few mutations in *DNAI2* gene have been described, supporting the hypothesis of an evolutionarily conserved functional role for *DNAI2* in ODA assembly [21].

Our case adds further information to PCD disease as result of alterations in *DNAI2*, corroborating the fundamental role of DNAI2 protein.

Interestingly, since the proband shows a less common clinical sign of PCD, such as hydrocephalus, in addition to the typical symptoms, our finding could add significant insights into genetic causes of hydrocephalus. Hydrocephalus is a common disorder of cerebral spinal fluid that has been widely reported in animal models with PCD, mainly in mouse [19], indeed, it has been showed that *Mdnah5*-deficient mice develop severe hydrocephalus at early postnatal ages, that is associated with ultrastructural axonemal defects within the ODA [11] and altered motility of ependymal cilia, so indicating a clear link between hydrocephalus formation and cilia dysfunction. On the contrary, there are few reports reporting hydrocephalus as clinical manifestation in human PCD subjects: the low prevalence of hydrocephalus in PCD patients suggests that distinct genetic mechanisms involved in the development and physiology of human and mouse brains are different. In literature, hydrocephalus has never been reported linked to *DNAI2* mutations, that is the novel finding here reported. In a few PCD cases described with hydrocephalus, ODA absence was the most frequent ultrastructural alteration observed [5, 32], even if some authors have also reported IDA defects, sometimes accompanied by a disorganization of the central-pair microtubules [1, 15, 28, 33]. In our case, a ODA absence or shortening in 100% of the ciliary cross-sections was detected by the electron microscopic examination and this result is consistent with an isolated ODA defect, in agreement with the genetic results.

However, the observation of NPH in this patient results very intriguing. NPH is a form of hydrocephalus characterized by ventricular enlargement and normal cerebrospinal fluid pressure. The aetiology of NPH is unknown and, to date, the genetic origin has been supposed in a few cases [12, 25, 29]. Therefore, the novel *DNAI2* microdeletion detected in our patient could suggest the potential implication of this genetic variant in the pathogenesis of NPH offering new insight on the genetic factors that could play a key role in the pathogenesis of NPH. Indeed, the mutations that compromise the function of motile cilia could be likely causative of the abnormalities in the flow of cerebrospinal fluid.

A major limitation of our case report is the lack of an expression assays or functional analysis; missing HSVMA study and nNO results are another limitation.

In conclusion, this study emphasizes further the important function of DNAI2, suggesting the alteration of DNAI2 protein as causative of PCD and as genetic risk factor of NPH. Furthermore, the study underlines the utility of application of new high-throughput technologies in order to get an early genetic diagnosis.

However, although the clinical manifestations of our patient suggest a likewise loss of function of DNAI2 protein, functional studies and immunofluorescence analyses are to be performed in order to better delineate the effect of the detected *DNAI2* deletion on ciliary ultrastructure.

## Supplementary Information

**Additional file 1.** 36 genes include in PCD panel.

## Data Availability

The reference sequence for validation of the deletion in the *DNAI2* gene was acquired from the NCBI Nucleotide database by using accession number NM_023036.6. The raw sequencing data is available in NCBI’s BioProject under the accession number SRR12830850, BioProject: PRJNA669228 (https://dataview.ncbi.nlm.nih.gov/object/SRR12830850)].

## Abbreviations

PCD: Primary ciliary dyskinesia
NPH: Normal Pressure Hydrocephalus
TEM: Transmission electron microscopy
nNO: Nasal nitric oxide
HSVMA: High-speed video microscopy analysis
*DNAH5*: Dynein Axonemal Heavy Chain 5
*DNAI1*: Dynein Axonemal Intermediate Chain 1
*DNAI2*: Dynein Axonemal Intermediate Chain 2
FUS: Foetal ultrasonography
CRP: C-Reactive Protein
WBC: White blood cell
NGS: Next Generation Sequencing
aCGH: Array Comparative Genomic Hybridization
ODA: Outer dynein arm
