# Supplementary material for: A novel genetic variant in DNAI2 detected by custom gene panel in a newborn with Primary Ciliary Dyskinesia: case report
Source: BMC Med Genet. 2020 Nov 10;21:220. doi: 10.1186/s12881-020-01160-5 (PMC7654168; doi:10.1186/s12881-020-01160-5)
Supplement: Supplementary file 1 — Additional file 1. 36 genes include in PCD panel. [file 12881_2020_1160_MOESM1_ESM.docx]

List of genes included in the panel:

CCDC39 (NM_181426), CCDC40 (NM_017950), RSPH9 (NM_0011933), RSHP4A (NM_001010892), RSPH3 (NM_031924), DNAJB13 (NM_163614), DNAH11 (NM_001277115), HYDIN (NM_001270974), CCDC65 (NM_033124), MCIDAS (NM01190787), RSPH11 (NM_080860), DRC1 (NM_145038), GAS8 (NM_001214), CCNO (NM_021147), DNAH5 (NM_001369), DNAI2 (NM_023036), DNAI1 (NM_012144), NME8 (NM_016616); DNAL1 (NM_031427), ARMC4 (NM_0180769), CCDC114 (NM_144577), CCDC151 (NM_002743), TTC25 (NM_031421), LRRC6 (NM_012472), DNAAF1 (NM_178452), DNAAF2 (NM_018139), DNAAF3 (NM_0012567414), DYX1C1 (NM_130810), CDC103 (NM_001258396), ZMYND10 (NM_015896), HEATR2 (NM_017802), SPAG1 (NM_172218), C21ORF59 (NM_021254), PIH1D3 (NM_173494), DNAH1 (NM_015512), STK36 (NM_015690).
